# Bridging the language barrier gap in the health of multicultural societies: report of a proposed mobile phone-based intervention using Ghana as an example

**DOI:** 10.1186/s40064-016-2602-x

**Published:** 2016-06-27

**Authors:** Judith Ansaa Osae-Larbi

**Affiliations:** Department of Psychology, School of Social Sciences, College of Humanities, University of Ghana, P. O. Box LG 84, Legon, Accra, Ghana

**Keywords:** Multiculturalism, Health, Language barriers, Mobile phone intervention, USSD codes

## Abstract

Across the globe, societies are recording an increasing number of domestic and international migrants for numerous reasons. While this may promote multiculturalism, new migrants and linguistically minority ethnocultural groups may face challenges in fully and equitably participating in various aspects of broader societies, due to language barriers. The purpose of this paper is to propose the use of Unstructured Supplementary Service Data (USSD) codes as an innovative intervention to address this long standing issue of language barriers, specifically as it pertains to accessing pertinent health information in multicultural societies. The USSD is a protocol that allows two-way communication between mobile phones and service providers, and which can be used independent of internet access. By dialing specific USSD codes (e.g. *555#) on their mobile phones, the proposed intervention would enable culturally and linguistically diverse populations in Ghana to access pertinent health information, particularly preventive information in understood language options on their phones. Using the current state of multiculturalism in Ghana as an example, this paper also describes how the proposed intervention can be developed, implemented and evaluated. The paper concludes by highlighting the significance of the proposed intervention to multiculturalism in Ghana and the implications for research. Bridging language barriers in access to health information is central to promoting the health of multicultural societies and fostering multicultural relationships. Therefore, overall, it is expected that this paper would stimulate thinking and research into innovative approaches that may help to successfully bridge language barriers in the area of health of multicultural societies.

## Background

Is there any contemporary society or country in the world comprised of a population, where all members are of the same cultural or ethnic group, speak the same language, and share a single cultural or civic identity? While the answer may be clear—none—the multicultural state of most contemporary societies readily brings to the fore, the problem of language barriers and their potential grave consequences across various sectors of the economy of these societies. In the area of healthcare, this problem has for many years been one, if not the greatest, barrier to effectively accessing and utilizing health information and services. This is particularly the case for new migrants from culturally and linguistically diverse backgrounds (Lee 2003).

This paper is a further development of a poster presentation,^1^ and seeks to propose the use of Unstructured Supplementary Service Data (USSD) codes as an innovative mobile phone-based intervention in response to this long standing issue of language barriers, as it pertains to accessing pertinent health information in multicultural societies. Specifically, the paper presents a brief overview of the benefits of language bridging in the area of healthcare; the challenges to using medical interpreters; the role of technology in language bridging; and the current state of multiculturalism in Ghana. Within this context of multiculturalism in Ghana, the paper further describes how public health stakeholders and telephone service providers can collaborate to develop, implement, and evaluate the impact of the proposed intervention on access to pertinent (preventive) health information. The paper finally concludes by highlighting the role of the proposed intervention in promoting multiculturalism in Ghana and the implications for research. Overall, the goal of this paper is to stimulate thinking and research into innovative and widely applicable ways of bridging language barriers that affect the health of multicultural societies.

### Benefits of language bridging in the health of multicultural societies

Across the globe, the benefits of removing language barriers in healthcare cannot be underestimated. Evidence indicates that it increases access to healthcare, promotes higher quality and safe care, improves patient satisfaction, enhances appropriate utilization of healthcare resources, and increases preventive health activities (Jacobs et al. 2004; American Institutes for Research 2005; Schyve 2007). Furthermore, improved physical and mental health through better access to health information and services typically underpins the day-to-day functioning of individuals and whole societies.

In some countries such as Canada, medical interpreters have been relied on to address the health information needs of migrants and non-dominant ethnocultural groups, who face language difficulties in healthcare settings (Lee 2003). Most studies assessing the benefits of language bridging in healthcare (e.g. Jacobs et al. 2004) have also focused on the effects of interpreter services. Indeed, reviews of such studies have shown that interpreter services improve important outcomes such as patient satisfaction, health care delivery, communication, and healthcare utilization (Karliner et al. 2007; Ramirez et al. 2008). A review by Bauer and Alegría (2010) has also shown that assessing patients’ mental health status in their non-primary language or using untrained interpreters can lead to inaccurate diagnosis. On the other hand, it was found that the use of professional interpreters may improve disclosure in patient-provider communications, referral to specialty care, and patient satisfaction.

### Challenges to the use of medical interpreters

While evidence supports the use of medical interpreters to improve access to and utilization of health information, it remains the case that these trained personnel are woefully scarce in most developing countries like Ghana. This may be due to the huge cost generally involved in training and hiring interpreters across healthcare settings (Dowbor et al. 2015). Even in countries with multiculturalism polices that foster bilingual competencies (e.g. Canada) and other developed countries such as Australia, the United Kingdom, and the United States, reports indicate a lack of professional interpreters (Dowbor et al. 2015; Lee 2003). These reports further suggest that linguistically minority groups in these countries may still face vital challenges to meeting their health information needs due to language barriers. In Ghana, where trained interpreters may be available, they may be fluent only in the languages of neighboring regions or countries. Furthermore, they may be primarily based in physical healthcare and urban settings and almost absent in mental healthcare and rural settings. Also, being typically based in hospitals, medical interpreters may not support easy access to preventive information among general populations faced with language barriers. Thus, for countries like Ghana, where the lack of healthcare resources necessitates primary prevention approaches to promoting public health, innovative solutions independent of real-time medical interpreters are worth considering.

### The role of technology in language bridging in multicultural societies

Over the years, various technological methods have been developed to meet the demand for innovative approaches to the problem of language barriers in access to healthcare information. The Integrated Healthcare Communicator (Cheong 2014) is one such approach. It is believed to be the first smartphone app to support voice translation of healthcare instructions from English into a local dialect (Cantonese). Real-time over-the-phone interpretation services have also been adopted in certain countries like Canada to support patient-provider communication in hospitals (Dowbor et al. 2015). Overall, a review of published literature and unpublished data on the use of language interpretation approaches indicate that technological advances are feasible in improving healthcare communication as well as quality of care for linguistically minority populations (Masland et al. 2010). Unfortunately, the typical internet and smartphone-dependent nature of many of these methods make them less applicable to developing countries like Ghana. In Ghana, majority of residents in the rural areas typically have limited access to internet and smartphones. Besides, similar to medical interpreters, most of these technological approaches can be used only within healthcare settings, limiting their use in promoting access to preventive healthcare information among Ghana’s general population.

Indeed, attempts are being made by some telephone service providers in the country to make health information economically accessible to customers. Basically, customers call their service provider’s health experts at reduced call rates. While this may provide convenient access to health information, there are several limitations. First, these services are limited in access to customers of respective service providers. Even among customers of such telephone network providers, not all may be aware of these services. Also, the health information may generally be provided in English. Further, the content of the information may be developed without much consideration of the unique health information needs of ethnocultural and migrant groups resident in different areas of the country. In effect, there exist a huge public health challenge of poor access to pertinent health information, particularly preventive information, among migrants, linguistically minority groups, and ethnocultural groups residing in Ghana.

### Ghana’s context of multiculturalism

Ghana is a West African country bounded by three francophone countries; on the north by Burkina Faso, on the east by Togo, and on the west by Côte d’Ivoire. Currently, the ten-region country is home to an estimated 75 ethnocultural/native groups, each speaking at least one distinct language, although some languages (e.g. Twi) may be commonly spoken by other cultural groups. Each region of the country is also home to internal migrants from other regions and international migrants from countries including Mali and China. Demographically, ideologically, and linguistically therefore, Ghana is a multicultural nation. Yet, despite her rich diversity, Ghana lacks formal multicultural policies, practices, and strategies necessary to ensure that individuals, irrespective of tribe, region, or country of origin have equitable rights to participate in, as well as benefit from the various sectors of the country’s economy.

In the area of health, the closest policy is the National Migration Policy for Ghana (Government of Ghana 2014), which recommends “the adoption of a framework to mitigate potential public health risks from migration, without adversely impacting the positive gains of migration.” (GoG 2014, p. 4). Although this policy is a positive step forward towards the protection of the health of Ghana’s multicultural societies, practical measures and strategies are needed to achieve its recommendations. As emphasized by Sam (2015), multiculturalism transcends the mere presence of diversity and policies. It is in actual sense, “the existence of, and a policy with its attending practices regarding the living together of, many ethnocultural groups in a plural society, as well as the normative beliefs that characterize how the relationships should be among the groups.” (Sam 2015, p. 3). In the absence of multicultural health methods in Ghana, it is not uncommon to see instances in hospitals where relatives, healthcare workers, and sometimes other patients are called on to serve as lay interpreters for patients facing language barriers. New migrants may also face significant challenges in gaining access to pertinent health information (e.g. about vaccinations) needed to protect their health and that of natives.

The following section duly proposes an innovative mobile phone-based strategy in response to the problem of language-barriers in accessing vital health information in Ghana. The section further discusses how the country’s multicultural regions/societies can collaborate with service providers to develop, implement, and evaluate the proposed intervention. In the light of Ghana’s new NMP, the proposed approach is indeed, timely for the country.

## Proposed intervention in the context of multiculturalism in Ghana

### Intervention description

The proposed intervention is the Unstructured Supplementary Service Data (USSD) multicultural health intervention service. The USSD is a protocol used by cellular phones to communicate with a telecommunication service provider. A USSD application has a unique short code in the form of *specific numbers# (e.g. *128#) or *specific numbers*specific numbers# (e.g. *121*2#). These short codes are dialed and sent to the service provider’s server using a phone. In response, the phone receives a message or a menu linked to different messages (from the server). Harnessing this two-way communication technology, the proposed intervention would allow culturally and linguistically diverse groups in the country to dial specific USSD codes on their phones, receive a menu of pertinent health information in response, and access (read) any information needed, in understood language options.

Among populations with limited use of smart phones, limited access to the internet, and/or limited access to important resources like banks and health information centers such as clinics, the USSD technology has been successfully used to bridge the gap in reaching these populations with vital services. Notable among these services are financial (mobile money) and bill paying services. In Uganda, this technology has also been used to successfully collect health research data among such populations (Namugaayi 2014). The use of USSD codes is independent of internet access, providing a wider reach to populations in rural areas and of all age groups.

### Intervention goal

The goal of the intervention is to support both local and international ethnocultural groups in Ghana to gain easy access to important health information. This includes information that promotes prevention of illnesses (e.g. malaria, cholera, Ebola, meningitis, hepatitis, diabetes, HIV/AIDs), encourages effective utilization of healthcare resources and services (e.g. free child immunizations, vaccinations, health screening), and facilitates navigation of healthcare processes and systems in Ghana (e.g. location and opening times of both public and private health facilities, available services in respective facilities, contact details of physical and mental health centers, standard referral procedures to larger hospitals or mental hospitals, as well as roles and contact details of social workers, community and lay healthcare workers, and relevant non-governmental health support organizations).

### Intervention stages

To achieve the above goal, it is proposed that a four-stage nonlinear model (hereafter, the Multicultural Health Information Model, MHIM) is used to guide the development and implementation of the USSD multicultural health information service. These stages are the “Understand”, “Collaborate”, “Innovate”, and “Evaluate” stages.

The MHIM was developed from prominent organizational change models including the Three-Step model of change (Lewin 1947); the eight-stage Action Research model (Lewin 1946); and the four-stage General Model of Planned Change (Cummings and Worley 2009). All three models generally direct change agents to first diagnose or reach detailed *Understanding* of the problem in need of change; collaborate with organizational members to plan and develop the intervention (*Collaborate*); implement the intervention (*Innovate*), and *Evaluate* any changes resulting from the intervention. Evidence supports the effectiveness of these models in guiding successful planned changes in organizations and whole systems, including medical/healthcare systems (Asumeng and Osae-Larbi 2015; Cummings and Worley 2009; Gallos 2006). Therefore, the MHIM, which is a comprehensive yet concise model from these tested models, may help to better promote efforts of Ghana’s public health system, specifically with regards to reaching culturally and linguistically diverse groups of people with critical health information. At each stage of the model, the following key actions may be necessary to bring about successful implementation of the USSD multicultural health information service.

### Stage one (understand)

At this preparatory and evidence gathering stage, three actions may be required. (1) The sponsoring (public health) agency overseeing the project such as the Ghana Health Service would determine the different languages that the health information may need to be translated into (e.g. English, Twi, Ga, Hausa, Ewe, Dagomba, French, Swahili, and Mandarin). (2) The agency would set up a committee in each region that may comprise of physical, mental, and public health professionals, social workers, lay persons from different migrant and ethnocultural groups of respective regions, research assistants, and certified interpreters for each language option. (3) Committees would diagnose the pertinent general and region-specific health information needs of ethnocultural groups in Ghana (e.g. vaccination requirements against meningitis for residents of the Northern regions) as well as collate information on the health resources and services available in each region using appropriate resources.

### Stage two (collaborate)

Here, two major actions may be required based on the understanding gained from the first stage. (1) Committees would develop content of the pertinent health information to meet the health general and region-specific health needs of ethnocultural groups. (2) Committees across the ten regions would work together to review and group information content in all languages and for each region into concise message sections that would be appropriately grouped under standard themes. Standard themes may serve as the menu from which specific information (sections) may be accessed. Here, any unique cultural values of the populations that speak the languages included in the multicultural health information service may be considered, for instance in how the information content may be presented or worded.

### Stage three (innovate)

Here the committees would work with all telecommunication service providers in Ghana to (1) Assign specific USSD codes to main health information menu options. (2) Assign navigation numbers to the different sections of information under each menu option. (3) Decide on the content of an automated prompt call and/or text message to be sent to all phones connected to a service provider in Ghana, as a strategy to raise and increase awareness of the proposed health information service. Prompt call messages would be repeated in all language options. (4) Decide on the times/frequency of disseminating the prompt message (e.g. at first connection of any phone to the server of any service provider in the country and every quarter to existing phone users). After completion of this stage, phone users can access pertinent health information in understood language options.

### Stage four (evaluate)

**Fig. 1:**
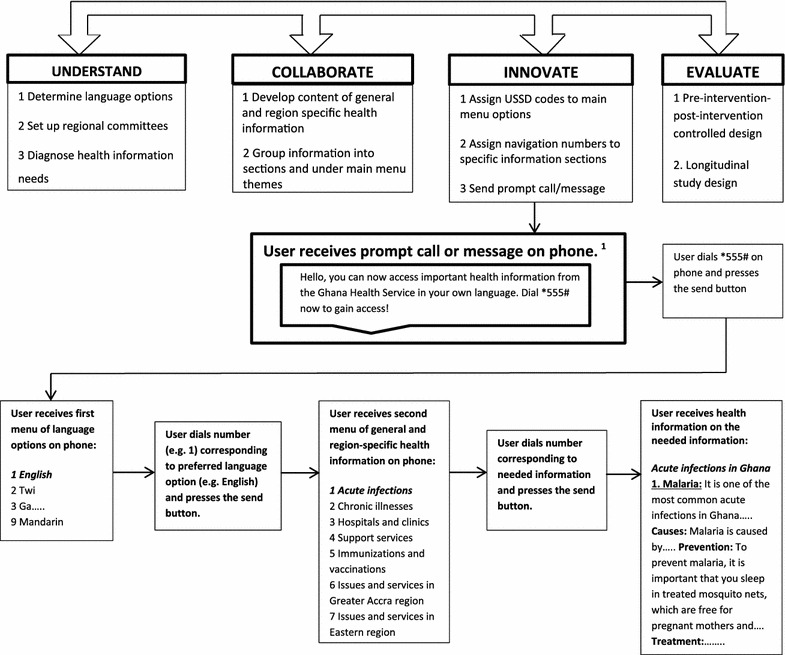
Proposed intervention development stages and example flow chart of the USSD multicultural health information service intervention. Beginning with the Understand stage, the intervention can be developed in four main stages that are non-linear, allowing intervention developers to revise the key actions that may be taken at any of the stages. The intervention will allow phone users to access pertinent health information on their phones by dialing a specific USSD code and following the navigation instructions to access the needed information in an understood language option. ^1^Prompt call message would be repeated in all the language options. Prompt (text) messages may be sent when user misses prompt call

The committees may decide on appropriate evaluation study designs at this stage or the first preparatory stage. Pre- and post-intervention (with control) data among linguistically diverse cultural groups across the regions may be collected to ascertain the effects of this innovation on level of access to key health information as a primary outcome. The influence of this innovation on physical and mental health status may also be assessed as long term outcomes in longitudinal study designs. Based on findings from the evaluation, committees can make appropriate revisions to actions taken at any of the preceding stages, as well as update content of the USSD multicultural health information service, in line with the non-linear nature of the MHIM. Figure 1 illustrates the proposed stages for the development of the intervention and how accessing a piece of health information may play out on a mobile phone.

## Discussion

This paper has proposed an innovative mobile phone-based technology—USSD codes—as a strategy to bridge the language barrier gap in accessing vital health information in multicultural societies. Using Ghana’s context of multiculturalism as an example, this paper has also described how relevant stakeholders can collaborate to develop, implement, and evaluate the impact of this phone-based intervention on important public health outcomes. Further, it has brought to the fore, the need for Ghana and similar societies to move beyond cultural diversity and establish policies, programmes, and practices that promote equitable access to health information. It is expected that successful development and implementation of the proposed language-bridging intervention would (1) improve easy access to well-understood critical health information for linguistically diverse cultural groups across Ghana; (2) promote the health of multicultural societies over time; and (3) inform successful ways of facilitating multiculturalism beyond the area of health, considering the centrality of effective two-way communication to all human interactions and institutions, particularly where cultural diversity is concerned.

Overall, it is expected that this intervention would help to foster multiculturalism in Ghana in a number of ways. First, successful implementation of this multilingual health information service may promote a sense of belonging to the wider Ghanaian society among culturally diverse groups, while indicating acceptance of the unique cultural and linguistic identities of these groups. Evidence of the effectiveness of the intervention (from the evaluation stage) may also encourage establishment of programmes to bridge language barriers in other aspects of Ghana’s economy. This may in turn, facilitate equitable participation of diverse cultural groups in the respective aspects of the country’s economy. Furthermore, resources that may have been used to manage health complications that may arise from poor access to preventive health information (due to language barriers), may be channeled into developing and improving multicultural programmes and practices. Additionally, the intervention may not only promote equitable access to pertinent health information, but also, improve the actual physical and mental health of ethnocultural groups in the country. This is fundamental to multiculturalism, as without health, there may be substandard and unequal participation by all culturally diverse groups in the country’s activities with its associated impaired intercultural relationship building.

### Limitations of proposed intervention

The ever evolving nature of language and healthcare needs, unsuitable collaboration/meeting times for committee members and other stakeholders, and the cost of developing accurate interpretations of health information content may prolong time for developing and evaluating the proposed intervention strategy. A cost-effectiveness evaluation study may however, help to ascertain the cost-effectiveness of the intervention.

## Conclusion and implications for research

In conclusion, language barriers may compromise the health of multicultural societies through poor access to pertinent preventive health information and its related poor access to quality healthcare services. Thus, all efforts to test the effectiveness and feasibility of the proposed language-bridging strategy, particularly in plural societies that lack multicultural health policies and programmes, are worth considering. It is imperative that longitudinal study designs are adopted to ascertain the long term impact of the proposed intervention on the health of migrant and ethnocultural groups, as well as the feasibility of the MHIM in guiding development and implementation of the intervention. Also, research that examines the impact of an extended version of the intervention, where the intervention content can be accessed as automated voice messages by dialing short codes on mobile phones, may support special groups such as the visually impaired and those incapable of reading, to access key health information. Finally, future research that considers the benefits of “global integration” as associated with the concept of omniculturalism, compared to the sociocultural integration of multiculturalism needs to be championed and any necessary revisions to the proposed intervention, duly effected.

## Footnotes

1 Poster presented at the 9th Biennial Conference of the International Academy for Intercultural Research (IAIR 2015), Bergen, Norway, 29 June–1 July, 2015.

## References

[CR001] American Institutes for Research (2005) A Patient-centered guide to implementing language access services in healthcare organizations. US Office of Minority Health. U.S. Department of Health and Human Services. http://minorityhealth.hhs.gov/Assets/pdf/Checked/HC-LSIG-ExecutiveSummary.pdf. Accessed 29 May 2016

[CR1] Asumeng MA, Osae-Larbi JA (2015). Organization development models: a critical review and implications for creating learning organizations. Eur J Train Dev Stud.

[CR2] Bauer AM, Alegría M (2010). Impact of patient language proficiency and interpreter service use on the quality of psychiatric care: a systematic review. Psychiatr Serv.

[CR3] Cheong K (2014) Hospital app that speaks in dialect. https://www.ihis.com. Accessed 29 May 2016

[CR100] Cummings T, Worley G (2009) Organization development and change, 9th edn. South Western, Cengage Learning Mason, USA

[CR4] Dowbor T, Zerger S, Pedersen C, Devotta K, Solomon R, Dobbin K, O’Campo P (2015). Shrinking the language accessibility gap: a mixed methods evaluation of telephone interpretation services in a large, diverse urban health care system. Int J Equity Health.

[CR101] Gallos J (2006) Organization development: a Jossey Bass reader, vol 4. Wiley

[CR5] Government of Ghana (2014) National migration policy for Ghana. Ministry of the Interior. Accra, Ghana

[CR6] Jacobs EA, Shepard DS, Suaya JA, Stone EL (2004). Overcoming language barriers in health care: costs and benefits of interpreter services. Am J Public Health.

[CR7] Karliner LS, Jacob EA, Chen AH, Mutha S (2007). Do professional interpreters improve clinical care for patients with limited English proficiency? A systematic review of the literature. Health Serv Res.

[CR8] Lee SM (2003). A review of language and other communication barriers in health care.

[CR9] Lewin K, Lewin GW (1946). Action research and minority problems. Resolving social conflict.

[CR10] Lewin K (1947). Frontiers in group dynamics II. Channels of group life; social planning and action research. Hum Relat.

[CR11] Masland MC, Lou C, Snowden L (2010). Use of communication technologies to cost-effectively increase the availability of interpretation services in healthcare settings. Telemed J e-Health.

[CR12] Namugaayi E (2014) How USSD is changing lives. https://www.thoughtworks.com. Accessed 29 May 2016

[CR13] Ramirez D, Engel KG, Tang TS (2008). Language interpreter utilization in the emergency department setting: a clinical review. J Health Care Poor.

[CR14] Sam DL, Moghaddam FM (2015). Multiculturalism. The SAGE encyclopedia of political behavior.

[CR15] Schyve PM (2007). Language differences as a barrier to quality and safety in health care: the Joint Commission perspective. J Gen Intern Med.

